# Clonal Signatures of Telomere Biology Disorders

**DOI:** 10.1007/s11899-026-00777-2

**Published:** 2026-04-14

**Authors:** Luca Arcuri, Emma M. Groarke, Sharon A. Savage, Fernanda Gutierrez-Rodrigues

**Affiliations:** 1 Hematology Branch, National Heart, Lung, and Blood Institute (NHLBI), National Institutes of Health (NIH), Bethesda, MD 20892 USA; 2 Hematology Unit, IRCCS Istituto G. Gaslini, Genova, 16147 Italy; 3 Clinical Genetics Branch, Division of Cancer Epidemiology and Genetics, National Cancer Institute, National Institutes of Health, Bethesda, MD 20892 USA

**Keywords:** Telomere, Telomere biology disorder, Clonal hematopoiesis, Somatic mutation, Myelodysplastic syndrome, Leukemia

## Abstract

**Purpose of Review:**

In inherited bone marrow failure syndromes (IBMFS), clonal hematopoiesis (CH) has been increasingly recognized as a molecular fingerprint of the underlying pathophysiology. A specific clonal profile has been reported in telomere biology disorders (TBDs), an IBMFS caused by pathogenic germline variants (PGV) in genes related to telomere maintenance and characterized by short/dysfunctional telomeres and increased risk of cancer. This review summarizes current data on specific somatic mutational profiles seen in TBDs and their associations with clinical features and cancer risk.

**Recent Findings:**

Recent studies have reported a specific CH landscape in TBDs that is associated with patients’ age, genotype, and phenotype. CH often involves the affected germline gene, with reversion or compensation of the PGV, or mutations in *PPM1D*, *TERT* promoter, or *POT1*—none of which are associated with increased risk of cancer. In contrast, a distinct group of recurrent CH that modulates TP53 pathway has been associated with cancer development in TBDs. These differing patterns of CH in TBDs have important implications for patients’ diagnosis, risk stratification, and surveillance.

**Summary:**

Characterization of clonal profiles across TBDs cohorts has helped identify potential molecular markers that can aid in diagnosis and guide future adapted surveillance and early intervention strategies. Although opportunities exist to incorporate CH into clinical care of patients with TBDs, multicenter longitudinal studies are still needed for validation and to allow for wide-scale adoption of CH into clinical care protocols.

## Introduction

Clonal hematopoiesis (CH) can be defined as the acquisition of somatic alterations in hematopoietic stem and progenitor cells (HSPCs) and their descendants with subsequent clonal expansion [1, 2]. The biological and clinical significance of these clones is influenced by several factors, including the affected gene, the mutation type, the underlying disease (if any), and the individual’s age [2]. CH has been increasingly recognized in inherited bone marrow failure syndromes (IBMFS), a group of disorders caused by pathogenic germline variants (PGVs) in genes related to maintenance, self-renewal, differentiation, and genomic stability of HSPCs [3]. In many IBMFS, CH reflects a molecular fingerprint of the underlying disorder characterized by disease-specific somatic mutational profiles not always driven by typical CH-associated genes and often independent of aging (e.g.,* EIF6* mutations in Shwachman-Diamond syndrome, *RUNX1* mutations in Fanconi anemia, and compensatory *SAMD9/9L* mutations in *SAMD9/SAMD9L* syndromes) [4–6].

CH in IBMFS can also be a mechanism of somatic genetic rescue (SGR) [7]; the acquisition of hematopoietic somatic mutations or chromosomal abnormalities may compensate for the effects of PGV on the intrinsic HSPCs restricted fitness. However, in some cases, a selective advantage of somatically mutated clones can lead to malignancy [4]. The effects of SGR can occur directly or indirectly on the germline affected gene (direct SGR and indirect SGR, respectively) [8]. Direct SGR is defined as the reversion or compensation of PGVs, most commonly by back mutation, loss of heterozygosity (LOH), copy-neutral loss of heterozygosity (CN-LOH), and large deletions or complete chromosome loss. Indirect SGR is defined as somatic alterations in genes related to pathways affected by the PGV [8].

Telomere biology disorders (TBDs) are a spectrum of cancer-prone IBMFS caused by PGVs in genes involved in telomere maintenance and integrity [9]. In this review we focus on direct and indirect SGR reported in TBDs, and their clinical potential.

## Telomeres and Cancer

**Fig. 1:**
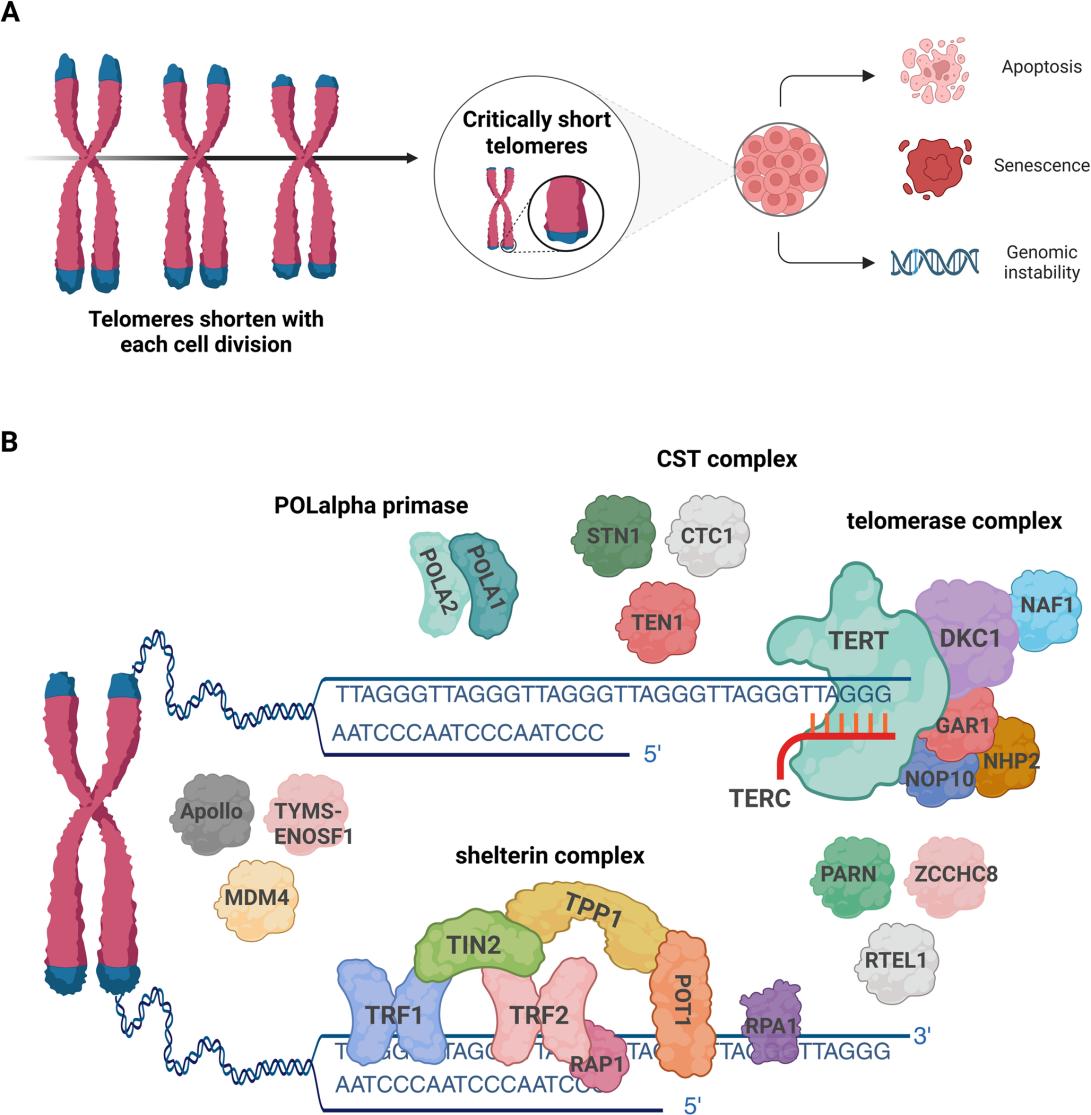
Telomere biology and structure.** A)** Telomeres shorten with each cell division. Critically short telomeres lead to genomic instability, triggering cellular apoptosis or senescence. Persistent cell division despite genomic instability can result in the accumulation of somatic mutations, which can lead to uncontrolled cell division and cancer. **B)** Schematic of the telomeres and associated proteins. Components are grouped approximately based on their function. Telomeres are located at the end of chromosomes and are composed of tandem TTAGGG repeats. Telomere-associated proteins, including telomerase, are critical for stability, integrity, and/or regulation of the telomerase complex, and telomere maintenance

Telomeres consist of long TTAGGG_(n)_ repeats and associated proteins located at chromosomal ends that are essential for genomic integrity. Telomeres prevent chromosome ends from being recognized as DNA double-strand breaks and inappropriate activation of DNA damage repair mechanisms [10]. Telomeres shorten with each cell division due to the inability of DNA polymerases to fully extend telomere ends (Fig. 1 A). Germ and stem cells express telomerase, encoded by *TERT*, a reverse transcriptase that extends telomeric DNA repeats using a RNA template encoded by *TERC*, thereby counteracting the natural telomere shortening occurring with each cell division [11, 12]. Additional genes encode proteins known to be critical for stability, integrity, and/or regulation of the telomerase complex, and telomere maintenance (Fig. 1B) [10, 13].

Telomere shortening acts as a tumor suppressor pathway, triggering cells’ senescence or apoptosis when telomeres are critically short [10, 14]. Cancer cells bypass these protective mechanisms, continuing to divide despite increased genomic instability. Telomerase upregulation also underlies tumorigenesis in most cancers, conferring cells uncontrolled proliferative advantage and accumulation of somatic mutations [14–17]. As result, telomerase has been targeted by many anticancer therapies with promising results by directly interacting with telomerase, or its components [18].

Abnormally short telomeres and increased risk of cancer are key features of TBDs.

## Telomere Biology Disorders

**Fig. 2:**
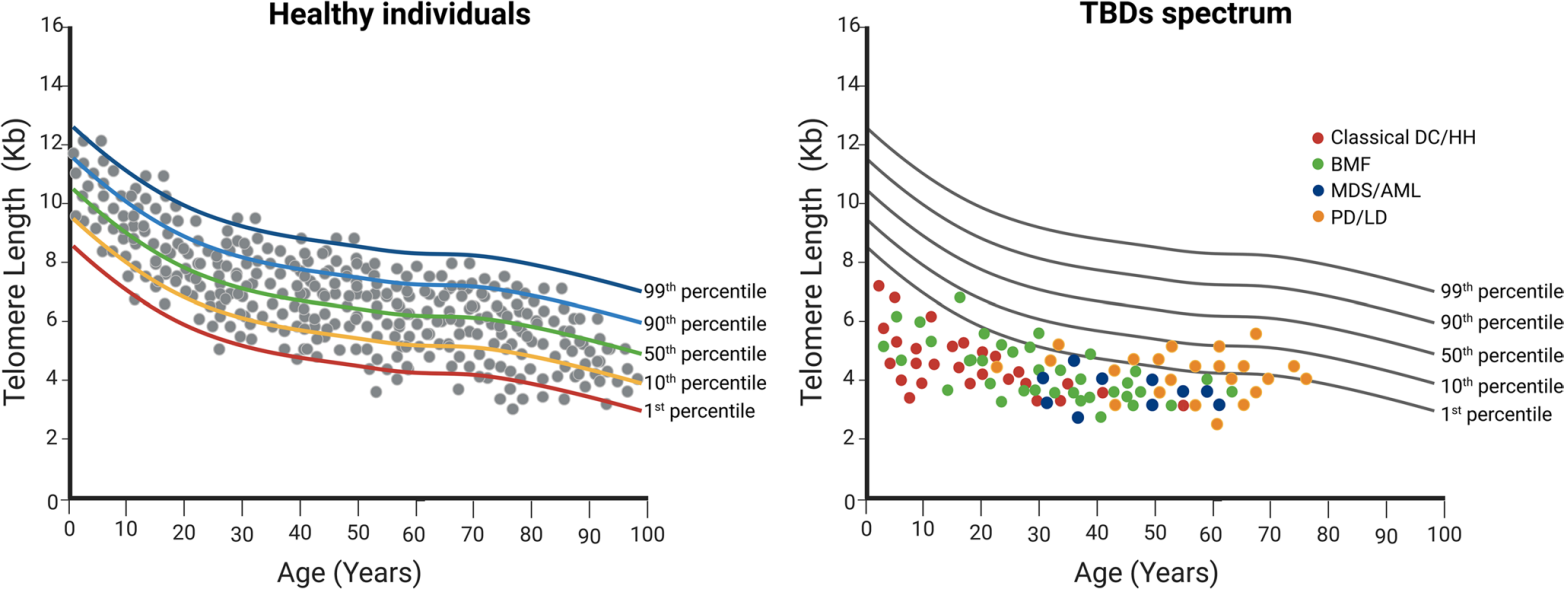
Representations of telomere length (TL) in healthy individuals and patients with telomere biology disorders (TBDs).** (A)** TL measurements in kilobases (kb) from lymphocytes of healthy individuals (grey circles) according to age, from birth (umbilical cord) to 100 years. In healthy individuals, TL ranges from approximately 11 kb at birth to 6 kb at 90 years of age. Percentiles fitted on TL measurements from healthy controls, as displayed in the figure, define the thresholds for TL shortening used in TBD diagnosis. The 50th percentile represents the median TL across age ranges. The 10th and the 1st percentiles indicate the thresholds below which 10% and 1% of TL measurements fall, respectively. The 90th and the 99th percentiles indicate the thresholds above which 10% and 1% of TL measurements fall, respectively. Very short and short telomeres are defined as those below the 1st and 10th percentiles of age-matched controls, respectively. **(B)** Representation of TL measurements in kilobases (kb) from lymphocytes of patients with TBDs and different phenotypes across a broad range of ages. Most patients with TBDs have very short or short TL; however, adult-onset TBDs, particularly those with pulmonary fibrosis, may present with normal TL (> 10th percentile of age-matched controls). Lymphocyte TL measured by flow cytometry with fluorescent in situ hybridization (flow FISH) <1st percentile for age is highly sensitive and specific for diagnosing TBDs. Abbreviations: DC/HH, dyskeratosis congenita or Hoyeraal-Hreidarsson Syndrome; BMF, bone marrow failure; MDS/AML, myelodysplastic syndromes and acute myeloid leukemia; PD/LD, pulmonary disease and liver disease

TBDs are a spectrum of disorders characterized by short and/or dysfunctional telomeres for age and a wide spectrum of manifestations with variable penetrance that present across all ages (Fig. 2). Lymphocyte telomere length measured by flow cytometry with fluorescent in situ hybridization (flow FISH) <1st percentile for age is highly sensitive and specific for diagnosing TBDs [9]. The clinical spectrum of TBDs primarily involves high rates of bone marrow failure (BMF), pulmonary and liver disease, and increased risk of hematologic and solid cancers as well as gastrointestinal, neurological, bone, vascular, and immune abnormalities [9, 19–23]. Dyskeratosis congenita (DC), the prototypical TBD, usually presents in childhood and is notable for the mucocutaneous triad of dysplastic nails, oral leukoplakia, and abnormal skin pigmentation, along with other TBD manifestations. Some individuals may develop phenotypes later in life with isolated features and telomeres < 10th percentile for age.

To date, TBDs have been attributed to PGVs in at least 17 genes related to telomere maintenance (Table 1) [10]. PGVs in additional genes have been associated with TBDs although their causal relationships remain incompletely established (*MDM4*,* NPM1*,* POLA1*,* POLA2*,* RPA2*,* SON*,* SHQ1*,* TYMS-ENOSF1*, and *USB1*) [10, 19, 24–28]. Disease onset and severity are associated with the affected gene and mode of inheritance: autosomal dominant (AD), autosomal recessive (AR), and X-linked recessive (XLR). Patients with AR/XLR and AD disease due to *TINF2* PGVs (separately classified as AD-*TINF2*) have severe phenotypes at younger age, and worse overall survival (OS) [29].

**Table 1:** Genes associated with telomere biology disorders with their inheritance mode and clinical associations

Gene	Pattern of inheritance	Clinical Phenotypes
*ACD*	AR[30], AD[30–33]	BMF[30, 31], HH[32], PD[33]
*CTC1*	AR[34–43], AD[44]	DC[34, 35], CP[36–43], PD[44]
*DCLRE1B*	AR[45]	DC[45], HH[45]
*DKC1*	XLR[46–60]	BMF[46], DC[47–57], HH[49, 56, 58–60], PD[46, 57]
*NAF1*	AD[61–63]	BMF[61], DC[61], PD[61, 62], MDS[61, 63], LD[61]
*NHP2*	AR[64–66], AD[64, 67]	MAA[67], DC[65, 66], HH[64], PD[64]
*NOP10*	AR[68], AD[69]	DC[68], PD[69]
*PARN*	AR[70–75],AD[76–82]	Isolated cytopenia[80], DC[71], HH[70, 72, 74, 75], PD[73, 76–82], MDS[72]
*POT1*	AR[24, 83], AD[7, 24, 84]	Isolated cytopenia[7], BMF[24], CP[83], PD[84], LD[7]
*RPA1*	AD[85]	DC[85], PD[85], MDS[85]
*RTEL1*	AR[86–97], AD[76–78, 90, 92–95, 98–103]	Isolated cytopenia[94, 100, 103], BMF[92–94, 103], DC[90, 97], HH[86–93, 96], PD[76, 77, 103, 104], LD[95, 100, 103], MDS[94, 95, 103]
*STN1*	AR[38, 105–108]	CP[38, 105–108]
*TERC*	AR[109, 110], AD[49, 56, 78, 80, 109, 111–124]	Isolated cytopenia[80, 124], MAA[124], SAA[124], BMF[49, 56, 80, 109, 117, 119], DC[49, 56, 109, 110, 112], HH[109], PD[78, 109, 111, 113–115, 118–120], LD[109, 115, 118, 120–122], MDS[49, 56, 80, 109, 115, 116, 119–121]
*TERT*	AR[109, 115, 125–131], AD[78, 104, 109, 114–116, 118–120, 126, 131, 132]	BMF[115, 116, 119, 131], DC[109, 116, 128], HH[109, 125–127, 129, 130, 133],PD[78, 104, 109, 114, 115, 118, 119, 131, 132], LD[78, 109, 115, 119, 120, 131], MDS[115]
*TINF2*	AD[134–147]	Isolated Cytopenia[136], BMF[136], DC[135–140], HH[136, 137, 147],RS [136, 137, 141–144], PD[134, 145, 146], MDS[146, 147]
*WRAP53*	AR[148–151]	DC[148, 149, 151], HH[150]
*ZCCHC8*	AD[24, 152–155]	MAA[155], BMF[24, 153], DC[24, 152, 153], HH[152], MDS[24, 153], PD[24, 153, 154], LD[155]

Abbreviations: *AR* Autosomal Recessive, *AD* Autosomal Dominant, *XLR* X-linked Recessive, *BMF* Bone Marrow Failure,* DC* Dyskeratosis Congenita, *HH* Hoyeraal–Hreidarsson Syndrome, *PD* Pulmonary Disease, *LD* Liver Disease, *MDS* Myelodysplastic Syndromes, *MAA* Moderate Aplastic Anemia, *SAA *Severe Aplastic Anemia, *CP* Coats plus Syndrome, *RS* Revesz Syndrome

Patients with TBDs have 3–4-fold higher risk of cancer than the general population, with some studies reporting genotype and/or mode of inheritance specificity [156–159]. The risk of hematological malignancies is overall markedly increased, with the risk of myelodysplastic syndromes (MDS) being 145 to 578-fold higher, and the risk of acute myeloid leukemia (AML) 21 to 74-fold higher than in the general population [157–159]. The most common solid cancers seen in TBDs are head and neck squamous cell carcinoma (SCC) [9, 29, 157, 159].

In TBDs, highest overall risk of cancer is seen in patients with AR/XLR disease [157]. Notably, patients with AR/XLR have the highest risk of developing solid cancers whereas those with AD-non-*TINF2* have the highest risk of hematologic malignancies. In our recent study of primarily AD genotype TBDs, cumulative incidences of MDS/AML and solid tumors at age 40 years were 8.6% and 3.6%, respectively [7]. Incidence of MDS/AML was higher in patients with PGVs in *DKC1*, *TERC*, and *TERT* (cumulative incidences of 17% for *DKC1*, 39% for *TERC,* and 22% for *TERT*) [7].

## Clonal Signatures of Telomere Biology Disorders

Both direct and indirect SGR have been linked to a specific clonal signature related to TBDs (Table 2). The reported CH landscape in TBDs is variable and dependent on multiple factors. Firstly, sequenced cohorts have had patient-related differences that may affect the CH profile, including age, genotype, and clinical phenotype. Secondly, technical factors, including sequencing detection limits and genes covered in the targeted panels have not been consistent. Nevertheless, many consistencies have been identified across different studies that warrant careful consideration. Here, we will focus on the clinical significance of direct SGR for diagnosis of TBD and recurrent indirect SGR for risk stratification and surveillance.

**Table 2:** Characteristics and clinical significance of clonal hematopoiesis in telomere biology disorders

SGR	TBD germline mutated gene	Inheritance mode	SGR type	Patient’s phenotypes at diagnosis	Functional Impact	Clinical significance in TBDs
Back mutation or CN-LOH	*TERC *[111, 160]	AD	Direct	DC[111], BMF[160], PD[111, 160], LD[160]	Restoration of the two wild-type alleles by direct reversion of the mutant nucleotide or loss of mutant allele followed by duplication of the wild-type counterpart.	Proof-of-pathogenicity of the telomere-related PGV. Often associated with improved blood counts or slow BMF progression, and TBD diagnosis in adulthood.
	*RPA1 *[85]	AD	Direct	DC		
	*ZCCHC8 *[152]	AD	Direct	DC		
	*DKC1 *[160, 161]	XLR	Direct	BMF[160], DC[161]		
Truncating *TINF2* mutations	*TINF2 *[7, 134, 162]	AD	Direct	BMF[162], DC/HH[7], PD[134]	Reduced expression of mutant allele and production of degenerate protein (in cis with the mutant allele)[134].	Strong evidence for PGV. May lead to atypical phenotypes other than classic DC/HH.
Loss-of-function *RPA1* mutations	*RPA1 *[85]	AD	Direct	DC	Compensates pathogenic gain-of-function *RPA1* PGVs by reducing the expression of mutant allele and production of degenerate protein (in cis with the mutant allele).	Strong evidence for PGV. Associated with stable blood counts over 20 years of follow-up.
Somatic *ZCCHC8* mutations	*ZCCHC8 *[152]	AD	Direct	DC	Likely compensatory CH. Not independently assessed from the co-occurring CN-LOH.	Strong evidence for PGV.
Somatic *TERT* promoter (*TERTp*) mutations	*TERT *[7, 132, 160, 163, 164]	AD[7, 160]	Direct	BMF[7, 160, 164], DC[7, 164], PD[7, 132, 160, 164], LD[7, 160, 164], asymptomatic[132, 164].	In cells from healthy people, *TERTp* increases *TERT*expression and cells’ proliferation, initiating tumorigenesis[16]. In TBDs, *TERTp* mutations are compensatory CH linked to increased telomerase activity and improved restricted fitness of *TERT *mutated HSPCs [132].	Increases the suspicion of an underlying TBD. Enriched in *TERT/TERC* patients. Associated with aging and multiorgan disease[7]. Associated with lower overall survival independent of age. Not associated with cancer development in TBDs.
	*TERC *[7, 160, 163, 164]	AD[7, 160, 164]	Indirect	BMF[7, 160, 164], PD[7, 160, 164], LD[7, 160, 164]		
	*CTC1 *[7]	AR	Indirect	BMF, PD, LD		
	*RTEL1 *[7, 160]	AD	Indirect	Asymptomatic[7], PD[160], solid tumor[160]		
	*NHP2 *[64]	AD	Indirect	BMF and PD		
	*NAF1 *[163]	AD	Indirect	Unknown		
	*PARN*[132]	AD	Indirect	PD		
Somatic *POT1* mutations	*POT1*[7]	AD	Direct	BMF, PD, LD[7]	Loss-of-function mutations that facilitate telomere elongation by allowing easier access to telomerase[163].	Increases the suspicion of an underlying TBD. Enriched in *TINF2* patients. Associated with improved cytopenia[162] or atypical and late-onset *AD-TINF2 *phenotypes[7]. Not associated with overall survival or cancer development.
	*TINF2*[7, 162]	AD[7, 162]	Indirect	BMF[7, 162], DC[7]		
	*TERT*[7, 163]	AD[7]	Indirect	BMF[7], PD[7]		
	*TERC*[7, 163]	AD^*7,*^	Indirect	BMF[7], PD[7], MDS[7]		
	*RTEL1*[7, 163]	AR[7]	Indirect	DC[7]		
Truncating *PPM1D* mutations	*DKC1*[7]	XLR	Indirect	DC	In cells from healthy people, truncating *PPM1D *mutations are negative regulators of p53 and reduce DNA damage response[165, 166]. In TBDs, *PPM1D* mutations are compensatory CH that appears to have limited capacity to confer cells proliferative advantage[7].	Not specific for TBDs, but part of a clonal profile that increases the suspicion of an underlying TBD. Associated with aging and multiorgan disease[7]. Not associated with overall survival or cancer development.
	*RTEL1*[7, 160]	AD[7], AR[160]	Indirect	Asymptomatic[7], BMF[160], PD[160]		
	*TERC*[7, 160]	AD[7, 160], AR[160]	Indirect	BMF[7, 160], PD[7, 160], LD[160], DC[160], MDS[7]		
	*TERT*[7, 160]	AD[7, 160], AR[160]	Indirect	BMF[7], PD[7, 160], LD[7]		
	*ZCCHC8*[160]	AD	Indirect	BMF, PD		
	*ACD*[160]	AD	Indirect	BMF, PD		
	*PARN*[160]	AD	Indirect	PD		
Loss-of-function *ATM * *mutations*	*TERC*[160]	AR, AD	Indirect	BMF, MDS, PD, LD	Likely compensatory CH with improvement of cell fitness by reducing DNA damage response-mediated senescence[160].	Unknown
	*TERT*[160]	AR	Indirect	BMF, MDS, PD, LD		
	*RTEL1*[160]	AD	Indirect	BMF, LD		
	*ZCCHC8*[160]	AD	Indirect	BMF, PD		
Somatic *U2AF1*^*S34*^ mutations	*TERC*[7, 160, 167]	AD[7, 160], AR[160]	Indirect	BMF[7, 160],PD [7, 160], LD[7, 160] Cytopenia[7], MDS[7, 160]	Pre-malignant CH that downregulates the TP53 and IFN pathways improving fitness of *TERT/TERC-*mutated HSPCs[7].	Not specific for TBDs, but part of a clonal profile that increases the suspicion of an underlying TBD. Associated with multiorgan disease, lower overall survival, and a ~ 30% risk of progression to MDS/AML 5 years after detection when secondary mutations in typical MDS/AML genes are also acquired[7].
	*TERT*[7, 160, 167]	AD[7, 160], AR[160]	Indirect	BMF[7, 160],PD [7, 160], LD[7], MDS[160], solid cancer[160]		
	*DKC1*[7, 160]	XLR	Indirect	DC[7], BMF[160], LD[160], MDS[7]		
	*PARN*[7]	AR	Indirect	DC		
	*CTC1*[7]	AR	Indirect	DC,CP		
	*RTEL1*[160]	AD	Indirect	BMF, Cytopenia, PD, LD, MDS, AML		
Somatic *TP53* mutations	*TERC*[7, 160]	AD	Indirect	BMF[7, 160], cytopenia[7],PD[7, 160], solid cancer[7], LD[160], MDS[160], AML[160]	Malignant CH that increases cells’ fitness bypassing p53 checkpoint controls[7, 163, 168, 169].	Not specific for TBDs. Associated with poor overall survival (< 20%) and a ~ 14% risk of progression to MDS/AML 5 years after detection. Associated with solid cancer development[7].
	*TERT*[7, 160]	AD	Indirect	BMF[7], Cytopenia[7], LD[160], MDS[160], AML[160]		
	*RTEL1*[7, 160]	AD	Indirect	BMF[7], Cytopenia[7], PD[7], LD[160], MDS[160], AML[7, 160]		
	*WRAP53*[7]	AR	Indirect	DC, solid cancer		
	*CTC1*[7]	AR	Indirect	CP		
	*PARN*[7]	AR	Indirect	Cytopenia		
	*DKC1*[160]	XLR	Indirect	MDS, AML, solid tumor		
Chr 1q gain (Chr1q+)	*TERC*[7, 160]	AD[7, 160]	Indirect	DC[7] AML[7], MDS[7]	Pre-malignant CH that downregulates TP53 pathways via *MDM4*[170].	Not specific for TBDs, but part of a clonal profile that increases the suspicion of an underlying TBD. Associated with multiorgan disease and a ~ 50% risk of progression to MDS/AML 5 years after detection when secondary mutations in typical MDS/AML genes are also acquired[7].
	*TERT*[7, 160]	AD[7, 160]	Indirect	BMF[7, 160], PD[7, 160], LD[7] AML[7], MDS[7, 160], solid cancer[160]		
	*RTEL1*[7, 160]	AR[7, 160]	Indirect	HH[7], LD[160], MDS[160], AML[160]		
	*PARN*[7, 160]	AD[160], AR[7]	Indirect	HH[7], BMF[160]		
	*ZCCHC8*[160]	AD	Indirect	BMF, PD		
	*TERC*[7, 160]	AD[7, 160]	Indirect	DC[7] AML[7], MDS[7]		
Partial or complete loss of chr7 (−7/7q)	*TERC*[7, 158]	AD[7]	Indirect	MDS/AML[7]	Malignant CH with haploinsufficiency of genes involved in myeloid tumorigenesis[171].	Not specific for TBDs. Marker of poor prognosis and indication for HCT[20, 172]
	*TERT*[7]	AD	Indirect	MDS/AML		
	*DKC1*[7]	XLR	Indirect	DC		

Abbreviations: *SGR* Somatic Genetic Rescue, *TBD* Telomere Biology Disorder, *DC* Dyskeratosis Congenita, *HH* Hoyeraal-Hreidarsson Syndrome, *BMF* Bone Marrow Failure, *PD* Pulmonary Disease , *LD* Liver Disease, *MDS* Myelodysplastic Syndromes, *AML* Acute Myeloid Leukemia, *CP* Coats Plus Syndrome, *CN-LOH *Copy-Neutral Loss of Heterozygosity, *CH* Clonal Hematopoiesis, *PGV* Pathogenic Germline Variant, *HSPC* Hematopoietic Stem and Progenitor Cells, *TERTp* TERT promoter, *HCT* Hematopoietic Cell Transplantation, *IFN* Interferon, *AD* Autosomal Dominant, *AR* Autosomal Recessive,* XLR* X-linked Recessive

### Direct SGR: Back Mutations, Uniparental Disomy, and Second-Site Mutations

Direct SGR ameliorates the restricted HSPCs fitness caused by PGVs at a cellular level, but this does not necessarily translate to a significant clinical improvement, such as telomere elongation or improvement of blood counts [8]. The reversion of PGVs to wild-type sequences by back mutation or restoration of two wild-type copies of the mutated gene by CN-LOH (or uniparental disomy; UPD) has been reported in multiple unrelated individuals with TBD harboring *TERC* and *DKC1* PGVs; these patients were usually older than typically observed, but still presented with very short telomeres, the mucocutaneous triad, and BMF [111, 160, 161]. These SGRs were noted due to unexpected allele frequencies of heterozygous PGVs in genetic testing; allelic imbalance was further confirmed by single nucleotide polymorphism (SNP) array [111, 160, 161].

Direct SGR in TBDs has had an important role in gene discovery as it can provide natural proof of the pathogenicity related to a PGV. For example, the recent identification of direct SGR in *RPA1* and *ZCCHC8* (Table 2) has expanded and confirmed the list of genes linked to TBDs [85, 152]. A *RPA1* PGV at VAF of 27% in BM but at 50% in fibroblasts due to UPD of chromosome 17 was detected in a patient with DC, who maintained stable blood counts and telomere length for over two decades [85]. More recently, two novel PGVs involving the *ZCCHC8* p.G184 codon were found etiologic in DC and associated with a large UPD of chromossome 12q encompassing the *ZCCHC8* locus [152]. Single-cell proteogenomic sequencing revealed that both 17p or 12qUPD in these cases were acquired in HSPCs but had myeloid bias during differentiation, with a variable fraction in lymphoid cells. Second-site mutations have also been reported in these *RPA1* and *ZCCHC8* patients (Table 2), supporting the evidence that these germline variants were pathogenic and related to BMF etiology.

Direct SGR by second-site mutations has been mostly reported in patients with *TINF2* and *TERT* PGVs (Table 2). Truncating somatic *TINF2* mutations were identified in three unrelated patients with *TINF2* PGV, two of whom had DC [7, 162] and one atypically presenting adult-onset pulmonary fibrosis [134]; in the latter, the somatic *TINF2* mutation, in cis with the *TINF2* PGV (located in the same allele), led to diminished expression of the mutant *TINF2* allele [134]. Direct SGRs in *TERT* are typically seen in the promoter region, discussed below.

### *TERT* Promoter and *POT1* Somatic Mutations as Direct and Indirect SGR

*TERT* promoter *(TERTp)* mutations at positions −146C > T, − 124C > T, and − 57A > C were first identified as a recurrent hit in many solid cancers, such as glioblastoma and melanoma [16, 173]. Functionally, these are known to activate *TERT* expression, upregulate telomerase, and confer cellular immortality to cells leading to malignant transformation [7, 16, 17, 132, 174, 175].

In TBDs, *TERTp* mutations are prevalent and associated with *TERT* and *TERC* genotypes (Table 2), occurring in 20–40% of patients with *TERT* (direct SGR) and 10–15% of those with *TERC* (indirect SGR); *TERTp* mutations are less frequent in patients with other TBD genotypes [7, 64, 132, 160, 164]. Similar to what is seen in cancer, *TERTp* mutations in TBDs are also at positions −124 C > T (60–80% of cases [7, 160, 163]), − 57A > C (25–35% of cases), and −146C > T (5% of cases) [7, 160]; found in *trans* with a germline TBD mutation, *TERTp* mutations are hypothesized to increase the proliferative advantage of TBD-mutated HSPCS by increasing the expression of the wild-type *TERT* allele [132]. Clinically, *TERTp* mutations have rarely been reported in pediatric TBD patients and have been associated with lower OS, independent of age [7]. *TERTp* mutations were enriched in patients with BMF and multiorgan disease, supporting the hypothesis that these mutations, poor prognostic markers for survival, are more likely to be selected in patients with progressive BMF, which in turn also correlates with multiorgan involvement and lower OS. In TBDs, *TERTp* compensatory effects in ameliorating hematopoiesis and improving blood counts may be limited by low rates of clonal expansion; *TERTp* mutations are generally found at very low VAFs (< 2%) in patients’ peripheral blood (PB). Though *TERTp* mutations have been associated with cancer in the general population, they have not been associated with an increased solid or hematological cancer risk TBDs. Interestingly, *TERTp* clones have been suggested to be protective for MDS/AML in TBDs [7, 160].

Another TBD-related gene involved in both direct and indirect SGR is *POT1* (Table 2). Similarly to *TERTp*, somatic *POT1* mutations initiate tumorigenesis in cells from healthy individuals and directly affect cells’ proliferation via telomerase [163, 176]. Somatic mutations in a specific domain of *POT1* critical for its binding to telomeres, and for the negative regulation of telomerase, have been reported in different human cancers including cutaneous malignant melanoma and chronic lymphocytic leukemia; telomere elongation rather than its shortening is a feature of these cancers [163, 177–181]. Somatic *POT1* mutations found in TBD patients are similar to those found in cancer and expected to increase cell proliferation and elongate telomeres. However, unlike in solid cancers, *POT1* mutations in TBD have not been associated with telomere elongation [7]. To date, there has been one case of a patient with a *TINF2* PGV treated with gonadotropin-releasing hormone (GnRH) agonist, who acquired two independent clones harboring somatic *POT1* and *TINF2* mutations. These mutations expanded during GnRH treatment, which coincided with an improvement in thrombocytopenia and elongation of telomeres in granulocytes and B cells [162].

In the setting of TBDs, *POT1 *somatic mutations have not been associated with increased risk of myeloid malignancy, solid cancer, or lower OS [7, 163]. Somatic *POT1* mutations were enriched in patients with *TINF2* hotspot (i.e., exon 6) PGVs; multiple *POT1* mutations at cumulative VAFs > 20% were detected in these patients, but not associated with cancer development. Instead, patients with AD-*TINF2* disease and a high burden of somatic *POT1* mutations demonstrated milder and late-onset phenotype of TBD with TL stability over the time in contrast to typical severe phenotype associated with this genotype [7].

### Indirect SGR in *PPM1D* and *ATM*

Somatic mutations in *PPM1D* have been reported in up to 30% of TBD patients (Table 2) and have not been associated with OS or increased risk of hematologic and solid cancer development, regardless of their VAF in PB [7, 160]. These mutations, detected at low VAFs (usually < 2%), were enriched in patients with *TERT* and *TERC* PGVs and often co-occurred with *TERTp* variants (26% of co-occurrence). *PPM1D* mutations found in TBDs are truncating, located in exon 6, and have been previously shown to negatively regulate p53 and attenuate the DNA damage response (DDR) in HSPCs [182]. Truncating *PPM1D* mutations have also been described in healthy individuals, particularly in those who had been exposure to genotoxic therapies or with shorter telomeres [2, 183–185]. In TBDs, *PPM1D* mutations have not been associated with previous chemotherapy. Telomere shortening does not appear to be the primary selective pressure since these are specifically enriched in *TERT* and *TERC* patients and not in the AR and *TINF2* genotypes which typically have the shortest TL.

Acquisition of *ATM* mutations has been reported in 9% of TBD patients (Table 2) [160]. In that study, most mutations were presumed to be loss-of-function and to attenuate DDR. Functionally, ATM inhibition counterbalances senescence driven by telomere dysfunction in fibroblasts of patients with telomere-related PGV [160]. The clinical significance of these mutations in TBD, including potential links to malignancy, is not well understood.

Similar to *TERTp*, the incidence of *PPM1D* and *ATM* mutations in TBDs appears to be age-dependent, being rare in children, and also correlates with the presence of BMF and lung or liver involvement [7]. Clones harboring *PPM1D* and *ATM* mutations are highly skewed toward the myeloid lineage [7, 160]. Clinically, there is no evidence, to date, that these mutations ameliorate patients’ clinical phenotypes or elongate telomeres. Recurrent selection of multiple *ATM* and *PPM1D* clones, a consequence of their emergence in independent HSPCs, are suggestive of strong selective pressure driven by dysfunctional telomeres.

### Development of Malignancy in TBDs Involves CH that Deregulates TP53 Pathway

Chromosome 1q gain (Chr1q+) and somatic mutations in *U2AF1* (particularly in the p.S34 hotspot) and in *TP53* were recently reported to be high-risk CH part of a canonical route of MDS/AML in TBDs that downregulates the aberrant TP53 activation typical of TBD-mutated HSPCs (Table 2) [168, 169]. *U2AF1*^*S34*^ mutations also downregulate the aberrant activation of interferon (IFN) pathways in HSPCs harboring *TERT/TERC* PGVs that leads to HSPC exhaustion [7, 170, 186]. Single-cell mutational trajectories and longitudinal monitoring of TBD patients with high-risk CH demonstrate two distinct patterns of malignant transformation: a progressive and slow process driven by either *U2AF1*^*S34*^ and Chr1q+ that often leads to MDS at an older age, or an unpredictable pattern driven by *TP53* mutations that often leads to MDS/AML evolution regardless of age.

Chr1q + and *U2AF1*^*S34*^ mutations are pre-malignant CH that initially appear to compensate for TBD HSPCs restricted fitness but ultimately drive the MDS/AML development when secondary mutations in typical MDS related genes (e.g. *RUNX1*, *ASXL1*,* SETBP1*,* ETV6*) are successively acquired in HSPCs in a linear trajectory [7]. Cumulative risk of MDS/AML in TBDs was approximately 50% and 30% 5 years after the detection of Chr1q + and *U2AF1*^*S34*^, respectively. However, the latency period from the appearance of Chr1q + and *U2AF1*^*S34*^ mutations to development of MDS/AML was variable and depends on the linear acquisition of second hits; in some patients, Chr1q + and *U2AF1*^*S34*^ were stable at high VAFs in PB without development of malignancy in over 15 years of follow-up. These alterations have been mostly observed in *DKC1* and *TERC* patients and are also associated with older age and multi-organ disease involvement.

In contrast, the acquisition of *TP53* mutations in patients with TBD has been associated with malignancy, irrespective of VAF in PB at first screening. They rarely co-occurred with other high-risk somatic alterations. The cumulative incidence of MDS/AML 7.5 years after first detection of *TP53* mutations was > 75%. *TP53* mutations were also associated with evolution to AML upon *TP53* biallelic inactivation (detection of chromosome 17p loss) [7]. While OS of patients with Chr1q + and *U2AF1*^*S34*^ mutations was approximately 50% at 5 years post their detection, OS of patients with *TP53* mutations was markedly worse, less than 25%. Interestingly, in the case of patients who acquired high-risk CH, most deaths were not directly due to cancer progression but included pulmonary fibrosis, infection, and complications of hematopoietic cell transplantation (HCT).

Interestingly, detection of *TP53* somatic mutations in PB was significantly associated with history of solid cancer in TBD patients. While the reasons underlying this are unclear, perhaps, *TP53* alterations in blood cells represent a convergent mechanism driving malignant transformation across different tissue types, supported by a recent study that found *TP53* somatic mutations in nearly all SCC tissues from TBDs patients [187].

Finally, monosomy 7 is a non-specific high-risk cytogenetic alteration reported at variable frequencies in different TBD cohorts (Table 2), and in both immune-mediated and inherited bone marrow failure [158, 163, 172]. High prevalence of ring sideroblasts in a TBD cohort with MDS/AML has also been reported but not described in other cohorts [158].

High-risk CH associated with MDS/AML is rare in pediatric TBD patients, correlating with a lower incidence of hematologic malignancy in these patients compared to adults. In our series, only one of 66 pediatric cases developed MDS, which coincided with detection of Chr1q + and *RUNX1* mutation at 7 years of follow-up; the patient had AR disease due to biallelic *PARN* PGVs. *U2AF1*^S34^ and *TP53* mutations were detected in two other pediatric cases that had not developed malignancy at last follow-up (at the age of 20.5 years and 18.7 years, respectively).

## Surveillance and Future Perspectives

Large, prospective longitudinal studies focused on identifying clinically significant predictors of cancer risk in TBDs are required to develop evidence-based screening guidelines and prevention strategies. Based on current evidence, there are opportunities to incorporate CH into clinical care of patients with TBDs: (1) to support the diagnosis of TBD, particularly in the context of clinical suspicion but without detection of a pathogenic or likely pathogenic TBD-related germline variant (either having a variant of uncertain significance or no variant), and (2) to stratify patients at high risk for development of myeloid malignancy and to guide follow up, including bone marrow surveillance strategies.

Direct SGRs are largely compensatory events that can serve as molecular markers of underlying telomere dysfunction. Patients with direct SGR can present with TBD germline variants at somatic ranges in PB and atypical and milder phenotypes compared to what is usually seen in the severe AR/XLR and AD-*TINF2* genotypes, though still associated with very short telomeres in most cases. Therefore, TBD diagnosis should not be prematurely ruled out when patients’ phenotypes are not classical of TBDs or when patients clinically suspected of TBD have negative germline genetic testing. Of note, approximately 20% of TBDs cases have unknown genetic defect. Characterization of direct SGR may require further genetic testing using a non-hematopoietic tissue such as fibroblasts. Isolated indirect SGR in TBD genes is not specific for TBDs, as it has been reported in both solid and hematologic cancers. However, detection of specific clonal patterns – for example, multiple *PPM1D* or *ATM* mutations, or co-occurrence of these with other recurrent CH such as *TERTp*, *POT1*, *U2AF1*^*S34*^, and Chr1q+, has potential to be clinically useful as these increase the suspicion of an underlying TBD in patients where there is diagnostic ambiguity.

In terms of malignant evolution, CH conveying high risk of MDS/AML should be closely monitored, and surveillance strategies adapted to account for specific genotype and age associations, and distinct patterns of malignant transformation driven by Chr1q + and *U2AF1*^S34^ vs. *TP53* mutations. In our cohort, 75% of CH considered high-risk was detected at initial evaluation (diagnosis or referral), by BM examinations with karyotype analysis and PB-targeted sequencing (limit of detection of 0.5%); high-risk CH absent at first evaluation but detected during follow-up was more likely observed in adults patients with *TERC* or *DKC1* PGVs or AR genotypes. Regarding age associations, only 5% of pediatric patients had high-risk CH at their first visit and developed malignancy at age < 18 years, highlighting the rarity of hematologic malignancy in this population. Our single pediatric case of MDS progression developed Chr1q+ after 7 years of annual BM examinations, along with a *RUNX1* somatic mutation in PB, suggesting that non-invasive somatic testing in PB may have been used for increased surveillance instead of multiple BM examinations. However, this approach requires prospective assessment and validation. Monosomy 7 detected by BM karyotyping represents a clear indication for transplant regardless of timing of detection [20].

In contrast, high-risk CH is detected in around 15% of adult TBD patients at first screening and its detection highly predicts MDS/AML within 5 years of follow-up. Patients with initial negative somatic testing but with high-risk genotypes for MDS/AML (*TERC*,* DKC1*, and *TERT*) and AR disease were more likely to acquire pre-leukemic CH during follow-up and therefore may benefit most from longitudinal assessment to detect emerging clones. It is important to note that currently, data are lacking on the optimal approaches to management. However, based on current data, TBD patients with *TP53* mutations have the highest risk of progression to hematologic malignancy, followed by those with *U2AF1*^*S34*^ and Chr1q+; therefore, serial monitoring is recommended. *TP53* somatic mutations in PB were associated with solid malignancy in TBD; its detection should prompt consideration of increased organ surveillance to identify solid cancers at an earlier stage.

In conclusion, CH is an important adjunctive diagnostic tool in TBD assessment, assisting in the diagnostic work-up of atypical cases, as well as overall TBD-associated cancer risk. CH profiles, along with patient age and genotype, have potential to help guide risk-adapted surveillance. CH screening at the initial visit of patients with high-risk genotypes for MDS/AML, AR/XLR diseases, and older patients with multi-organ involvement should be strongly considered. Multi-center longitudinal studies across the age and genotype spectrum of the TBDs are required to establish the ideal timing of intervention to improve patients’ survival and/or quality of life.

## Key References

- Jongmans Marjolijn C, Verwiel Eugene T, Heijdra Y, et al. Revertant Somatic Mosaicism by Mitotic Recombination in Dyskeratosis Congenita. *American Journal of Human Genetics*. 2012 Mar 9;90(3) doi:10.1016/j.ajhg.2012.01.004.
  - ◌ First report of somatic reversion in TBD.
- Alder JK, Stanley SE, Wagner CL, Hamilton M, Hanumanthu VS, Armanios M. Exome Sequencing Identifies Mutant TINF2 in a Family With Pulmonary Fibrosis. *Chest*. 2015/05/01;147(5) doi:10.1378/chest.14-1947.
  - ◌ Fisrt report of second-site mutations in TBD.
- L M, Y Y, A Y, et al. Somatic mutations in telomerase promoter counterbalance germline loss-of-function mutations - PubMed. *The Journal of clinical investigation*. 03/01/2017;127(3) doi:10.1172/JCI91161.
  - ◌ First report of *TERT* promoter mutations in TBD.
- Gutierrez-Rodrigues F, Donaires FS, Pinto A, et al. Pathogenic TERT promoter variants in telomere diseases. *Genetics in Medicine*. 2018 Dec 7;21(7) doi:10.1038/s41436-018-0385-x.
  - ◌ Study that expanded the TBD phenotypes associted with *TERT* promoter mutations.
- Schratz KE, Gaysinskaya V, Cosner ZL, et al. Somatic reversion impacts myelodysplastic syndromes and acute myeloid leukemia evolution in the short telomere disorders. *The Journal of Clinical Investigation*. 2021 Sep 15;131(18) doi:10.1172/JCI147598.
  - ◌ First study that reported somatic *POT1* mutaions in TBD.
- Gutierrez-Rodrigues F, Groarke EM, Thongon N, et al. Clonal landscape and clinical outcomes of telomere biology disorders: somatic rescue and cancer mutations. *Blood*. 2024/12/05;144(23) doi:10.1182/blood.2024025023.
  - ◌ First study that reported specific CH as predictors of MDS/AML and survival in TBDs.

## Data Availability

No datasets were generated or analysed during the current study.
